# Culturally sensitive patient-centered healthcare: a focus on health behavior modification in low and middle-income nations—insights from Indonesia

**DOI:** 10.3389/fmed.2024.1353037

**Published:** 2024-04-12

**Authors:** D. A Cipta, D. Andoko, A. Theja, A. V. E. Utama, H. Hendrik, D. G. William, N. Reina, M. T. Handoko, N. Lumbuun

**Affiliations:** ^1^Department of Family Medicine and Primary Care, Faculty of Medicine, Universitas Pelita Harapan, Tangerang, Indonesia; ^2^Department of Psychiatry, Faculty of Medicine, Universitas Pelita Harapan, Tangerang, Indonesia; ^3^School of Medicine, Faculty of Medicine, Universitas Pelita Harapan, Tangerang, Indonesia

**Keywords:** patient-centered, culturally sensitive care, health behavior change, low and middle-income countries (LMICs), culture-specific patient empowerment, patient education, behavior modification techniques

## Abstract

Patient-centered, culturally sensitive healthcare acknowledges the profound impact of cultural beliefs on health behaviors and outcomes, particularly vital in low and middle-income countries (LMICs). Within Indonesia, distinct cultural factors are pivotal in empowering patients, necessitating their integration into healthcare practices. For example, the cultural concept of *gotong royong*, emphasizing communal collaboration, presents an opportunity to foster community support networks among patients. Moreover, honoring familial ties and involving family members in decision-making enhances patient empowerment. Acknowledging and incorporating spiritual and religious beliefs, which are deeply rooted in Indonesian culture, into healthcare interventions further augments patient empowerment and well-being. In LMICs, including Indonesia, achieving patient empowerment demands implementing critical strategies. Community-based interventions harness local resources and engage the community to drive health behavior change. Culturally sensitive communication bridges the gap between healthcare providers and patients, respecting language nuances and cultural norms. Patient education fosters a comprehensive understanding of health conditions, thereby encouraging active involvement in decision-making. Tailored behavior modification techniques, aligned with cultural beliefs and practices, support the adoption of healthier behaviors among patients. This review emphasizes the pivotal role of patient-centered, culturally sensitive healthcare in LMICs, particularly in Indonesia. It delves into strategies to promote health behavior change within these unique contexts, emphasizing the importance of cultural sensitivity and patient-centered care. The discourse also explores the cultural landscape impacting healthcare, acknowledging the challenges faced in delivering comprehensive healthcare services within these diverse cultural contexts. Additionally, it outlines innovative approaches and success stories in implementing patient-centered care, highlighting how cultural factors intersect with healthcare outcomes. By advocating for integrating culture-specific patient empowerment practices into healthcare methodologies, this article underscores the potential for improved health outcomes, heightened patient engagement, and the delivery of culturally relevant services within LMICs.

## 1 Introduction

Patient-centered, culturally sensitive health care prioritizes each patient’s cultural background, tailoring healthcare services to align with their individual needs. This approach holds particular significance in LMICs, where cultural beliefs and practices substantially influence health behaviors and subsequent outcomes. At the core of this paradigm lies the crucial significance of utilizing patient-centered, culturally sensitive strategies to accomplish enduring changes in health behavior (1, 2). This manuscript delves into the paramount importance of patient-centered, culturally sensitive healthcare within LMICs. Specifically, we explore diverse strategies to foster health behavior change within these unique settings.

LMICs face multifaceted challenges in delivering comprehensive healthcare to their populations. These hurdles encompass scarce resources, inadequate infrastructure, and the need for more trained healthcare professionals. Furthermore, cultural beliefs significantly impact health behaviors, decision-making, and health outcomes. For instance, Indonesian culture, though varied between tribes, religion, and living settings (urban vs. rural), usually places substantial emphasis on traditional medicine. This often leads to preference for traditional healers over Western medical practices (1, 2). Additionally, the culture promotes profound reverence for elders, who play significant roles in decision-making processes. It upholds communal decision-making, where local leaders, community elders, and family members play pivotal roles in treatment implementation and decision-making processes.

Recognizing the undeniable influence of cultural beliefs and practices on health behaviors and outcomes, patient-centered, culturally sensitive healthcare strives to integrate these factors into healthcare services. This is particularly crucial within primary care settings. Some evidence underscores the positive impact of this approach on patient satisfaction, treatment adherence, and overall health outcomes (2, 3).

## 2 Health behavior change

In general understanding, behavior is considered a determinant of health and is the target of promotion for behavior change. The process encompasses shifting from negative (unhealthy) behavior to positive behavior that aligns with health values, fostering the development or enhancement of positive behavior, and maintaining pre-existing positive behavior or behavior consistent with health norms and values (4–6). Emphasizing the preservation of already established healthy behaviors, this transformative journey acknowledges that an individual’s behavior can change when an internal imbalance exists within them (3, 4).

Several stimuli can lead individuals to change their behavior; social factors, as external influences on behavior, include social structures, social institutions, and other social issues (4). Factors influencing behavior change include the pre-existing personality, influenced by individual characteristics, assessment of offered changes, interactions with healthcare professionals recommending behavior change, past experiences attempting similar changes, and general cultural values that serve as either rewards, reinforcers, and/or punishers. For example, tobacco smoking for men is connected to cultural practice in many parts of Indonesia, while for women, it is just a relatively recent or urban practice, and the latter are often viewed quite negatively by the larger society. Stimuli originating from emotions such as fear and love or personal and cultural expectations can also influence behavior (7).

Behavior change usually takes time, and few individuals instantly alter their behavior. Sometimes, people change due to societal pressures or a desire to conform to existing norms (8). The process of change is not immediate and should be thoroughly tested. Prochaska and DiClemente’s model proposes a structured framework consisting of five stages in the behavior change process that provide a comprehensive and sequential understanding of the dynamic journey individuals undergo when striving for change, namely health behavior change (9).

The relationship between health and behavior is closely intertwined, with a healthy individual reflecting healthy behavior and vice versa. The crucial benefits of a healthy life include enhancing our productivity and utilizing our abilities and potential to the fullest. Therefore, the concept of healthy living, such as promoting Clean and Healthy Behavior (*Perilaku Hidup Bersih dan Sehat* or PHBS program in Indonesian Health Centers), needs to be nurtured by every individual to improve overall health quality. Healthy behavior and behavior change aim to establish a healthy lifestyle pattern that reflects positive habits (10).

In the health behavior change process, a significant aspect is the formation and alteration of behavior, serving as the objective of health education or health counseling to support other health programs. The intended changes go beyond and encompass covert behavior. Concrete and positive efforts are necessary within health programs to achieve behavioral changes aligned with health norms (9, 10).

Behavioral change strategies can be divided into three categories: utilizing power or incentives that lead to rapid but less sustainable changes, providing information that leads to enduring public awareness, and participatory discussion that leads to active participation resulting in more steadfast and profound changes (11). The effectiveness of these strategies may be further improved by tailoring them to the patient’s needs and culture (10).

Patient-centered, culturally sensitive healthcare is therefore vital for improving health behavior and achieving better health outcomes. The process may include various approaches such as education, counseling, and behavior modification techniques (BMT) (12). Unhealthy behaviors such as smoking, poor diet, and lack of physical activity are major risk factors for chronic diseases such as heart disease, diabetes, and cancer. Health behavior change is essential for prevention; modifying the risk of chronic diseases will improve overall health and well-being and is also important for the management of chronic diseases (12). For example, individuals with diabetes may need to modify their diet and exercise habits to manage their blood sugar levels. Health behavior change can also improve medication adherence and reduce the risk of complications associated with chronic diseases.

Education entails providing tailored information about health conditions and the significance of behavior change, utilizing visual aids based on the individual’s education level and intellectual development. Counseling involves collaborative efforts to identify and overcome barriers to behavior change, with healthcare providers or trained counselors offering support. Behavior Modification Techniques (BMT) utilize positive reinforcement, incorporating rewards and incentives to induce health behavior change. Techniques such as peer support groups and community networks foster an environment conducive to sustained behavioral shifts. These methods leverage incentives, such as facility access or health-related service discounts, promoting adherence to desired behaviors. Social support networks promote mutual encouragement and the exchange of experiences, reinforcing long-term commitment to behavior change. The combination of these techniques, incentives, and social networks offers a comprehensive approach to catalyzing enduring health behavior modifications effectively (13).

Facilitating health behavior change within LMICs presents intricate challenges rooted in cultural beliefs that diverge from Western medical practices. For instance, some cultures prioritize traditional healers over Western medicine, perceiving the pursuit of medical care as indicative of vulnerability. These entrenched cultural paradigms serve as substantial impediments obstructing efforts toward behavior modification (14). In this context, the significance of patient-centered, culturally sensitive healthcare surfaces as a pivotal determinant.

## 3 Implementation of culturally sensitive patient-centered healthcare to change health-related behavior: challenge and potential strategies

Despite the challenges, some strategies can effectively promote health behavior change in LMICs. These strategies include community-based interventions, culturally sensitive communication, patient education, and BMT (15, 16).

### 3.1 Community-based interventions

Community-based interventions are indeed effective in promoting health behavior change. A systematic review of community-based interventions found that these approaches successfully promoted physical activity, healthy eating, and tobacco cessation (17). Additionally, they have been shown to promote medication adherence and improve health outcomes in individuals with chronic diseases (18).

Community-based interventions are particularly effective when tailored to the community’s cultural background. For example, community-based interventions involving traditional healers have effectively promoted health behavior change in some cultures (19). Community-based interventions involving peer support groups and other social support networks have also effectively promoted health behavior change (20).

Posyandu (*Pos Pelayanan Terpadu*), or Integrated Health Posts, are widespread throughout Indonesian villages and suburbs. Efforts to revitalize Posyandu have focused on strengthening community involvement, providing additional training to health workers, and integrating new services, such as chronic disease management or mental health support, to better address the evolving health needs of communities. The establishment of Posyandu within communities, particularly in underserved regions, significantly fosters advancing health and familial well-being within a given locality. In this context, Posyandu must operate efficiently, as evidenced by data provided by the Ministry of Health of the Republic of Indonesia in 2018, which indicated the presence of 173,750 active Posyandu dispersed across Indonesia, boasting an operational engagement rate of 61.32% (21).

Even during the COVID-19 pandemic, Posyandu continued their activities by conducting mobile visits to the homes of toddlers, facilitated by the *kader*, a term used to describe community health workers (CHWs). In the existing system, these kader have not yet been officially incorporated into the healthcare system and therefore are not officially funded. Authors’ field experience and anecdotal reports suggest that *kader* are delegated by the local health service, often without their voluntary consent or prior training, yet they are expected to run Posyandu activities and mobilize the community. These activities include administering vitamin A supplementation, vaccinations, and providing education on complementary feeding, among others (22, 23).

In the domain of mental health, leveraging the influence of religious and grassroots community leaders to facilitate access to mental health professionals, such as psychiatrists and psychologists, has proven beneficial in reaching isolated patients and enhancing their outcomes. This approach aids in preventing further harm, such as instances of shackling and chronic disability. Collaborative efforts between family physicians and local kader are often necessary to implement these initiatives effectively. Furthermore, in Indonesia, individuals can access free preventive measures, consultations, and treatments through the national health insurance scheme, *BPJS Kesehatan*. This ensures that mental health services are accessible to all, regardless of socioeconomic status (24).

Additionally, kaders and primary care physicians conduct home visits to administer long-acting antipsychotic injections for patients with schizophrenia, ensuring continuity of care and adherence to treatment regimens. Training sessions offered to individuals with mental disorders (known as “ODGJ” or *Orang Dengan Gangguan Jiwa*) in essential life skills and productivity not only assist them in their daily routines but also engage the participation of their immediate social circles. This benefit aligns with the program’s goals, which aim to enhance the involvement of families and communities. Consultations for families of ODGJ, including discussions about the condition of the individuals, are also offered to increase understanding and empathy towards ODGJ, given the significant role that families play. This benefit is consistent with the program’s objective of increasing family involvement in mental health efforts (25).

Community-based interventions in Indonesia often intertwine health practices with religious beliefs to promote health and well-being. By recognizing and respecting the significance of these practices within the community, interventions can effectively engage community members and foster meaningful participation. For example, initiatives may collaborate with local religious leaders or traditional healers to integrate health promotion messages into religious gatherings or formal healing sessions. This approach ensures cultural relevance and acceptance and enhances the reach and impact of interventions by tapping into existing community structures and networks. Additionally, community-based interventions may facilitate dialogue and collaboration between healthcare providers and community members to develop culturally sensitive health programs that align with religious and cultural values, ultimately contributing to improved health outcomes at the community level (21, 22).

Indonesia, as the country with the largest Muslim population in the world, serves as a significant hub for Islamic culture and practices. With approximately 87% of its population adhering to Islam, Indonesia boasts a diverse and vibrant Muslim community that permeates various aspects of Indonesian society, including daily life, governance, cultural traditions, and health. In addition to serving as centers for health promotion and education, mosques in Indonesia often host free mass circumcision events for Muslims (26).

Additionally, efforts are made to portray it as promoting cleanliness and personal hygiene. These endeavors aim to provide scientific credibility and a moral basis for the practice. Because accumulations of urine and smegma beneath the foreskin can lead to impurity on clothing and the body, many Islamic scholars interpret circumcision legislation as a means to purify the body from such impurities. Further research has provided increasing evidence for the health benefits of circumcision, including a lower risk of Human Immunodeficiency Virus (HIV), Herpes Simplex Virus Type 2 (HSV-2), and Human Papillomavirus (HPV) infection. Among female partners of circumcised men, bacterial vaginosis was reduced by 40%, and Trichomonas vaginalis infection was reduced by 48%. Urinary tract infections in infants during their first year can pose serious risks, potentially requiring hospitalization. The likelihood of a urinary tract infection in an uncircumcised male infant is ten times higher than in a circumcised male infant, with rates of 1 in 100 and 1 in 1000, respectively (26).

In Indonesia, Friday prayer sermons often incorporate messages about health and hygiene, serving as platforms to encourage congregants to adopt healthy lifestyles and seek medical care when necessary. These sermons are vital in disseminating health-related information and promoting preventive healthcare practices within Muslim communities. Islamic teachings emphasize the importance of cleanliness and hygiene as integral aspects of faith and daily life. The Qur’an instructs believers to maintain cleanliness and purification, as stated in Surah Al-Ma’idah (5:6). Additionally, authentic Hadiths, such as those found in Sahih Muslim and Sahih Bukhari, emphasize the significance of cleanliness and regular ablution (wudu) before prayer. These teachings influence health behaviors among Muslims, promoting practices that enhance personal hygiene and contribute to overall well-being. The emphasis on cleanliness extends beyond physical rituals to encompass mental, spiritual, and environmental cleanliness, reflecting Islam’s holistic approach to health and hygiene (26).

Despite their effectiveness, community-based interventions face several challenges. These challenges include limited resources, inadequate infrastructure, and a shortage of trained healthcare providers. Additionally, cultural beliefs such as communities trusting traditional healers more than Western medicine are still prevalent in rural areas (27).

Some strategies can be effective in overcoming the challenges of community-based interventions. These strategies include working with community leaders. Community bonds and collective decision-making play a vital role in Indonesia. Implementing community-based health approaches that involve local leaders, community elders, and traditional healers can foster a sense of ownership and empowerment among patients. Community leaders can help to promote health behavior change and encourage community members to seek medical care when necessary (28).

Building rapport with non-health stakeholders, such as local government officials, school teachers, and community leaders (often religious leaders), is pivotal to addressing limited resources by seeking donations from stakeholders (27, 28).

Addressing limited infrastructure can involve setting up an integrated network of school clinics with the public health system or establishing Posyandu at local mosques. Additionally, utilizing technology, such as mobile phones and social media, can help overcome infrastructure barriers by providing health education, counseling, and other healthcare services to community members (27, 28).

The shortage of healthcare providers may also be alleviated by training community leaders and willing participants in the community to be CHWs, enabling health education to be delivered by prominent members in the community (28).

### 3.2 Culturally sensitive communication

Culturally sensitive communication operates within the broader framework of cultural sensitivity, which requires individuals to be aware of cultural diversity and its implications for a patient’s beliefs and attitudes, while also respecting individual differences (29). Cultural sensitivity is defined as ‘the ability to recognize, understand, and react appropriately to the behaviors of persons who belong to a cultural or ethnic group that differs substantially from one’s own (30). Healthcare communication, involving the exchange of information between patients and healthcare providers, extends to interactions with families and caregivers. This form of communication entails bi-directional engagement, involving patients in decision-making processes and care planning.

Culturally sensitive communication is essential in patient-centered, culturally sensitive healthcare. It involves tailoring health messages to the patient’s cultural background (27). This constitutes a fundamental aspect of healthcare delivery, as it can significantly impact the quality of care and patient and family satisfaction. When cultural disparities arise, they may lead to poor adherence to treatment, worse health outcomes, and an increased prevalence of adverse events (28).

The concepts of culturally sensitive communication involve three major parts: antecedents, attributes, and consequences. Antecedents are aspects that precede circumstances or events. In culturally sensitive communication, antecedents include the environment and culture of the ward, organizational structures, the clinician’s education and communication experience, sociocultural characteristics of patients, families, and clinicians, and the personal and professional experiences of the clinicians (29). The next concept is defining attributes, which involve communication between clinicians, patients, and families. It is divided into four attributes: encouraging patients and families to participate in decision-making, prioritizing cultural considerations in planning, developing a trusting relationship, and using personal interpreters where language differences exist. The last concept is consequences, which are incidents that occur due to culturally sensitive communication. Outcomes include increased patient and family satisfaction, improved adherence to treatment regimens, better engagement in patient and family-centered care, and improved health outcomes (31).

Indonesia is the fourth most populous country in the world, with an estimated population of 260 million people. It is also known as a culturally diverse country with more than 1,300 ethnic groups and six official religions. The multifaceted nature of health behavior in Indonesia can be attributed to the nation’s diverse ethnic composition and multicultural demographic, which includes various health providers. Despite the wide availability of formal health services, cultural or traditional health providers comprise a significant component of health services. Some examples in Indonesia include mind–body therapies, such as hypnotherapy, physical therapies with tools like acupuncture, physical therapies without tools such as body massage, and biologically based therapies using substances from nature (31).

This highlights the importance of culturally sensitive communication in LMICs. Health messages tailored to the patient’s cultural background are more likely to be understood and followed. Culturally sensitive communication can also help overcome cultural barriers to health behavior change. For example, in some cultures, seeking medical care may be viewed as a sign of weakness, leading individuals to prefer traditional healers over Western medicine. In instances such as the treatment of prevalent conditions like depression and anxiety, cultural perceptions emphasizing spiritual fortitude may inadvertently engender moral assessments and perpetuate stigmatization.

A culturally attuned clinician confronted with barriers, such as language, socioeconomic status, literacy, and occupation, must navigate them by integrating culturally resonant narratives and metaphors that align with prevailing cultural beliefs, while concurrently providing scientific rationale. This approach facilitates a bridge between cultural understanding and evidence-based medical practice, allowing for the effective prescription of routine care within culturally diverse contexts (32).

Culturally sensitive communication is effective in promoting health behavior change. A systematic review of culturally sensitive interventions found that these interventions were effective in promoting physical activity, healthy eating, and tobacco cessation. Using language and symbols familiar to the patient can help promote understanding and adherence to health messages. This may involve the use of local dialects and visual aids that are tailored to the cultural background of the patient (32).

Several strategies can be implemented to facilitate culturally sensitive communication. Awareness of one’s own culture, including an understanding of one’s own cultural beliefs, attitudes, values, and practices, is crucial. This is particularly significant in Indonesia, with its diverse ethnic composition that requires greater interpersonal cultural awareness associated with patient and family satisfaction. Clinicians are more likely to deliver personalized and culturally sensitive care to patients by enhancing their comprehension of diverse cultures, including values, attitudes, and beliefs. This initial step necessitates self-awareness to mitigate the risk of overgeneralization and stereotyping of cultures (32).

Establishing open and sensitive communication is essential, incorporating active listening and respect for an individual’s cultural beliefs and practices. This approach fosters a therapeutic relationship built on trust and respect. It also aids in collaborating on treatment strategies with the patient and family in decision-making regarding healthcare (32).

Another strategy involves prioritizing cultural consideration in the planning and provision of care. Clinicians can achieve this by asking culturally sensitive questions about the patient’s and family’s values, beliefs, and practices. This includes exploring their beliefs associated with the presenting illness and assessing the individual’s psychological and sociocultural needs, such as secondary languages, religion, and food preferences (32).

### 3.3 Patient education

Patient education is important for promoting health behavior change. Patients who are informed about their health condition and the importance of health behavior change are more likely to take an active role in their healthcare and make positive changes to their health behaviors. Patient education can also help to overcome cultural barriers to health behavior change. For example, the coexistence of traditional healers and Western medicine doctors in Indonesia reflects the country’s rich cultural and healthcare landscape. Both systems play significant roles in addressing the health needs of the population, including patient education. People often navigate between the two based on accessibility, personal beliefs, cultural preferences, and the nature of their health conditions. In some cases, collaboration between traditional healers and Western medicine practitioners is becoming more common, especially in addressing chronic conditions or complex health issues such as diabetes, hypertension, maternal mortality, and promoting health behavior change (33).

Research conducted regarding culture-based patient education among the Makassarese People in South Celebes, Indonesia, found that integrating educational materials into the local Makassarese language and modifying intervention programs to suit local culture, for example, using local produce and foodstuffs, has been effective in diet modification and reducing blood sugar levels in people with diabetes (34). Storytelling methods, such as using Wayang (Indonesian traditional puppets) and folk stories, also effectively promoted tobacco cessation, drug abuse prevention, and a healthy lifestyle in adolescents (35). Another study about collaborative programs between local health services and community and religious leaders in Central Java has also shown to promote medication adherence and improve health outcomes in individuals with diabetes mellitus (36).

Using visual aids, such as diagrams and charts, can enhance the understanding of health messages, especially in rural societies with low literacy rates. Health communication in the local language is crucial for effective communication. In Indonesia, utilizing local languages in health communication materials can bridge the communication gap and empower patients to better understand their health conditions and treatment options (37).

Tailoring patient education to the patient’s needs, by using language and symbols familiar to the patient and presenting information in a format that is easy to understand, promotes better understanding and adherence to health messages. Additionally, tailoring education to the patient’s culture by designing health education materials that are culturally relevant to the Indonesian context can improve health literacy and empower patients to make informed decisions about their health (37).

Providing ongoing patient education and support is vital for reinforcing health messages and promoting long-term behavior change. This may involve follow-up visits, telephone calls, or text messages. Another strategic approach is the use of social media, given its pervasive reach across urban and rural landscapes. Leveraging social media involves collaborating with influential figures who serve as role models for promoting healthy lifestyles. Engaging these influencers in advocating and exemplifying healthful behaviors enhances visibility and fosters credibility and resonance among the younger demographic, significantly influencing health behavior modification initiatives (37).

An essential consideration for clinicians involves being attuned to patients’ comprehension after disseminating medical information. Cultural perception is a significant filter, and in our clinical experience, neglecting to assess patient understanding can yield detrimental outcomes. Certain terms, such as ‘*cuci darah*’ (hemodialysis), may evoke considerable fear, leading to the refusal of crucial life-saving treatments. Moreover, initial resistance, particularly regarding invasive procedures or interventions affecting reproduction in women, often necessitates a nuanced approach. Decision-making processes frequently involve the individual, their male partners, and extended family, highlighting the collective nature of decision-making in contrast to the Western perception of autonomy. Addressing these dynamics requires a dialectical approach within clinical interactions (38).

### 3.4 Behavior modification techniques

Behavior Modification Techniques (BMT) are an essential component of health behavior change. They involve using rewards and incentives to promote positive health behavior change (13). BMT is important for promoting health behavior change, particularly for behaviors such as smoking, poor diet, and lack of physical activity, which are often difficult to change. BMT can provide incentives and rewards that motivate individuals to positively change their health behaviors, and they can also help overcome cultural barriers to health behavior change. For example, in some cultures, seeking medical care may be viewed as a sign of weakness, and traditional healers may be preferred over Western medicine. BMT can help overcome this barrier, by giving incentives to motivate the community to change their views (13, 39).

Facilitating the establishment of social support is another effective strategy. Social support through peer support groups and other social networks can reinforce positive health behaviors and incentivize behavior change. In our experience, empowering patients and their families to join a group with a similar context, not necessarily a similar diagnosis, and facilitating them to lead and support each other has proven effective. A clinician-centered group may not be sustainable in the long run, and patient and family-led groups are often more effective based on our observations (39).

Utilizing technology, such as mobile phones, social media, and disease-specific web-based or mobile applications, can provide incentives and rewards for behavior change. Technology can offer reminders, feedback, and other incentives for positive health behaviors. For instance, a web-based application has been developed to assist patients, families, and cadres in monitoring the symptoms of schizophrenia and ensuring medication adherence. Another mobile application was also found to be effective in promoting physical activity, healthy eating, and tobacco cessation (39).

## 4 Discussion

Patient-centered, culturally sensitive healthcare is essential in healthcare delivery, particularly in low- and middle-income countries (LMICs). This literature review highlights the importance of patient-centered, culturally sensitive healthcare in promoting health behavior change in LMICs. Health behavior change is a critical aspect of healthcare delivery, especially in LMICs with a high burden of preventable diseases. It refers to adopting healthy behaviors and ceasing unhealthy ones. Patient-centered, culturally sensitive healthcare can promote health behavior change in LMICs by addressing cultural barriers to behavior change.

Healthcare providers need to understand and appreciate the cultural diversity of their patients to provide appropriate care that addresses cultural barriers to behavior change. Studies have shown that culturally appropriate health education and counseling are associated with improved health behavior change. Healthcare providers who understand and appreciate cultural beliefs can provide appropriate health education and counseling that resonates with patients’ cultural beliefs. This can lead to improved patient satisfaction, adherence, and health outcomes.

Future research endeavors should prioritize the development of culturally tailored health education and counseling interventions to drive health behavior change within LMICs. A crucial aspect involves crafting interventions that align with patients’ cultural beliefs, practices, and values. Active engagement of patients and community members in formulating these interventions is fundamental. Culturally appropriate interventions bear the potential to foster meaningful health behavior changes, thereby contributing to enhanced health outcomes. Additionally, research efforts should explore optimal methodologies for training future clinicians, especially within the primary care sector, spanning undergraduate, postgraduate, and continuing medical education (CME) frameworks.

## 5 Conclusion

Patient-centered, culturally sensitive healthcare is an approach that considers the patient’s cultural background and tailors healthcare services to meet the patient’s needs. This approach is particularly important in low- and middle-income countries (LMICs), where cultural beliefs and practices can significantly impact health behaviors and outcomes. Strategies that can effectively promote health behavior change in these settings include community-based interventions, culturally sensitive communication, patient education, and behavior modification techniques.

## Data availability statement

The original contributions presented in the study are included in the article/supplementary material, further inquiries can be directed to the corresponding author.

## Author contributions

DC: Conceptualization, Writing – original draft, Writing – review & editing, Project administration, Supervision. DA: Conceptualization, Writing – original draft. AT: Conceptualization, Data curation, Project administration, Writing – original draft. AU: Conceptualization, Writing – original draft. HH: Conceptualization, Writing – original draft. DW: Supervision, Writing – review & editing. NR: Project administration, Writing – review & editing. MH: Conceptualization, Project administration, Writing – review & editing. NL: Conceptualization, Supervision, Writing – review & editing.
